# An Overview of Respiratory Syncytial Virus

**DOI:** 10.1371/journal.ppat.1004016

**Published:** 2014-04-24

**Authors:** Jia Meng, Christopher C. Stobart, Anne L. Hotard, Martin L. Moore

**Affiliations:** 1 Department of Pediatrics, Emory University, Atlanta, Georgia, United States of America; 2 Children's Healthcare of Atlanta, Atlanta, Georgia, United States of America; Columbia University, United States of America

## Respiratory Syncytial Virus (RSV) and Child Health

Respiratory Syncytial Virus (RSV), a member of the Paramyxoviridae family, is the leading cause of lower respiratory tract illness (LRI) in infants. From 1993 to 2008, the total RSV hospitalization rate in the United States across all age groups was 55 per 100,000 person-years, slightly lower than the rate of 64 per 100,000 person-years for influenza viruses [1]. In infants, the hospitalization rate was 2,345 per 100,000 person-years for RSV compared to 151 for influenza, consistent with reports that RSV hospitalizes 1–2% of infants in the US each winter, a staggering statistic [1]. RSV disease is not limited to infants. RSV resulted in more hospitalizations in 1–4-year-olds than influenza [1]. One in 13 children under the age of five in the US required medical attention for RSV each year, and 60% of office visits were for 2–5-year-olds [2].

RSV remains a significant cause of death. Over all age groups, influenza caused three times as many deaths as RSV in the US from 1990 to 1999, mostly in the elderly [3]. RSV caused 137 deaths per year in the US in children less than 4 years old, compared to 38 per year in this age group for influenza [3]. Globally, RSV was estimated to have caused 66,000 to 199,000 pneumonia deaths in children less than 5 years old in 2005, making RSV the third most important cause of deadly childhood pneumonia after *Streptococcus pneumonia* and *Haemophilus influenza* [4]. RSV is increasingly recognized as a global health priority.

Other than ribavirin, there are no licensed RSV vaccines or therapeutics. The monoclonal antibody (mAb) palivizumab is a neutralizing mAb against a conserved epitope in the viral fusion (F) surface glycoprotein. Palivizumab is administered prophylactically to high-risk infants, such as those with chronic lung disease of prematurity, congenital heart disease, or premature birth at less than 36 weeks gestational age, but it costs $4,500 per patient treatment course [5].

## RSV Pathogenesis

RSV apically infects ciliated epithelial cells of the airways. RSV bronchiolitis is characterized by mucus in the airways, sloughed epithelial cell debris, and abundant neutrophils. Airway mucus is a hallmark of RSV LRI, contributing to pulmonary obstruction, but mechanisms of RSV-induced mucus expression remain unclear. RSV-induced mucus is a particular problem in the small diameter airways of premature infants.

Mice are semipermissive for RSV replication. Though more permissive than other inbred mouse strains, BALB/c mice infected with commonly used laboratory RSV strains (e.g., A2 or Long strains) do not exhibit high viral loads or pulmonary mucus. Mouse models, though mechanistic, likely overestimate immune-mediated pathology in RSV pathogenesis. The picture from infants is that fatal RSV LRI is a virus-induced pathology, not an exaggerated immune-mediated pathology [6]. Cotton rats are more permissive and exhibit more RSV antigen in the airway epithelium than mice, but cotton rats also lack lung pathology characteristic of RSV disease.

Our laboratory developed RSV strains that are relatively more pathogenic than commonly used laboratory RSV strains in mice and recapitulate airway mucus and respiratory compromise. We generated a recombinant, strain-chimeric RSV strain, A2-line19F, which expresses the F gene of the mucus-inducing line 19 RSV strain in the genetic background of the laboratory A2 strain [7]. A2-line19F induces airway mucus and exhibits higher viral load in mice than laboratory strains, implicating RSV F in mucus induction [7]–[9]. Breathing difficulty (retractions and nasal flaring) is a symptom of RSV bronchiolitis. One way to quantify breathing effort is to measure pulsus paradoxus, the distension of arteries in the periphery due to breathing effort. RSV A2-line19F and the clinical isolate RSV 2–20 increased breathing effort in mice [8], [10].

## RSV Immunity

Immunity to natural RSV infection is partial but protective. Symptomatic RSV reinfection in early childhood is common. Homologous RSV strains can reinfect persons of all ages; thus, sterilizing immunity is not established. However, repeat infections are associated with decreased risk of LRI even if the secondary infection occurs in the first year of life [11]. Thus, in contrast to the lack of solid immunity to RSV upper respiratory tract illness (URI), protective immunity to RSV LRI builds rapidly. This provides rationale for RSV vaccines in the target population aimed at preventing disease. In one study, 64% of infants younger than 9 months old developed neutralizing antibodies (nAb) after primary RSV infection [12].

T cells are important for RSV clearance. In infants, CD8 T cells in the airways correlate with recovery, not disease [13]. In mice, protective versus pathologic roles of T cells depend on the model system. CD8 T cells are critical for RSV clearance, can mediate weight loss and lung lymphocytic inflammation in primary infection, and protect against immunopathology associated with failed vaccines. Vaccine-elicited CD8 T cells can protect against RSV challenge and against RSV A2-line19F-induced mucin expression [9].

**Figure 1:**
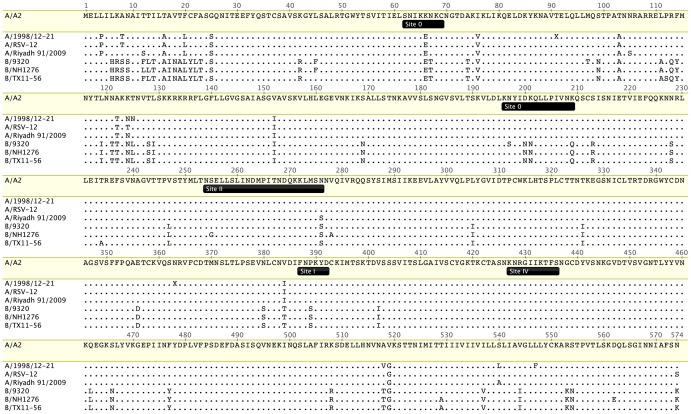
Conservation of RSV F protein antigenic sites characterized to date. Four antigenic group A and three group B RSV F protein sequences were aligned using ClustalW and visualized with Geneious Pro software. The RSV A2 F (Genbank protein accession ACO83301) serves as the reference strain. The other representative strains are A/1998/12-21 (Nashville, Tennessee, US, accession AFM95385), A/RSV-12 (Denver, Colorado, US, 2004–2005, accession AEO45929), A/Riyadh/2009 (Riyadh, Saudi Arabia, accession AEO23052), B/9320 (Massachusetts, US, 1977, accession AAR14266), B/NH1276 (New Haven, Connecticut, US, 2002, accession AFD34264), and B/TX11-56 (Dallas, Texas, US, 2011, accession AFD34265). Positions of antigenic sites 0, I, II, and IV are from reference [15].

A good correlate of protection for RSV is nAbs (mucosal IgA and serum IgG via transudation). There is one serotype of RSV and two antigenic groups, A and B. RSV A is more prevalent and slightly more pathogenic than RSV B. Seroconversion rates (based on neutralization) were reported to be highly group-specific, so a bivalent vaccine may be optimal [14]. Nevertheless, the RSV F protein is relatively conserved (**Figure 1**), and some anti-F neutralizing mAbs such as palivizumab can protect against A and B strains.

Known nAbs to RSV are specific for either F or the attachment (G) glycoprotein. RSV G is heavily glycosylated and antigenically heterogeneous. Anti-G mAbs are generally less neutralizing in vitro than anti-F mAbs, so F is the preferred vaccine antigen. RSV F and G are both important targets for in vivo neutralization, with F playing a somewhat greater role based on studies in experimental animals. Like F proteins of model paramyxoviruses, RSV F undergoes a conformational change from a prefusion to a postfusion form. Unlike model paramyxoviruses, the RSV attachment glycoprotein is not required for F triggering or virus replication in vitro. Recently, prefusion and postfusion structures of RSV F were solved [15]. An antigenic site (site 0) specific for prefusion F was localized to the top of the prefusion head. Site 0 is targeted by novel mAbs with higher potency than palivizumab [15], [16]. Prefusion F-specific Abs are prevalent in polyclonal neutralizing antisera [17]. However, antigenic site 0 is less conserved than site II to which palivizumab binds (**Figure 1**).

## RSV Inhibits Host Immune Responses

The RSV nonstructural-1 and -2 (NS1 and NS2) proteins inhibit innate and adaptive immune responses to RSV. NS1 and NS2 have multiple functions. For example, NS1 antagonizes type I interferon, dendritic cell maturation, and T cell responses [18]. NS2 binds RIG-I and potently degrades STAT-2 [19], [20]. RSV G is immunomodulatory by at least two mechanisms. RSV produces a secreted G (sG) form in abundance that serves as an antigen decoy, similar to the Ebola virus secreted glycoprotein [21]. Also, a chemokine motif conserved in G modulates immune responses [8].

## RSV Vaccines and Antivirals

The challenges to RSV vaccine development are substantial [22]. In 1969, a formalin-inactivated RSV plus alum adjuvant (FI-RSV) vaccine resulted in severe disease exacerbation upon natural RSV exposure. FI-RSV has fettered RSV vaccine development in the past. The FI-RSV vaccination+RSV challenge-enhanced disease immunopathology phenotype is reproducible across laboratories, animal models, and related viruses in their natural host, such as bovine RSV and pneumonia virus of mice. However, mechanisms of FI-RSV-enhanced disease appear multifactorial and remain to be fully elucidated. F and G subunit vaccines studied early on have a history of disease enhancement, albeit to a lesser extent than FI-RSV. RSV subunit vaccines have never been tested in naïve infants. RSV live attenuated vaccines (LAV) have a good safety record in infants.

In RSV naïve infants, the path is paved for RSV LAVs. The advantages of LAVs for other viruses are well known. RSV was attenuated by classic forward mutagenesis. Using reverse genetics, attenuating mutations were incorporated into RSV in different combinations in attempts to strike a balance between attenuation and immunogenicity. A recent RSV LAV candidate (rA2cp248/404/1030ΔSH) was safe in infants but poorly immunogenic, as measured by serum Ab [23]. The Collins group at the National Institute of Allergy and Infectious Diseases (NIAID) and MedImmune have explored a range of attenuation and immunogenicity in a series of RSV A2 strain mutant LAVs, but the optimal balance has proven elusive. Actually, the total number of RSV mutants published is very low compared to other important viruses, owing in part to the small size of the RSV field and technical challenges of RSV reverse genetics [24]. The achievable RSV attenuation and immunogenicity remains to be fully explored. For example, targeted mutagenesis of RSV virulence genes could enhance immunogenicity. Another issue with RSV LAVs is the genetic instability (reversion) of point mutations derived by classical mutagenesis. Again, novel mutations via reverse genetics may elucidate stable attenuation.

Similar to influenza, tetanus, and pertussis, one strategy to target RSV by vaccination is maternal immunization. There is significant RSV disease in 0–2-month-old infants, who will be difficult to protect by active immunization but could be protected by boosting maternal nAb. Critical issues are the half-life of the transferred Ab, estimated to be 1 month, the timing (3rd trimester) of immunization, and insufficient Ab transfer in the case of premature birth.

Antivirals are being developed against RSV. One example is a RSV nucleoside analog against the viral polymerase (Clinicaltrials.gov identifier NCT01906164). One paradigm is that usefulness of antivirals for RSV in infants will be limited because viral load has peaked by the time of hospitalization. However, in a careful study of RSV clearance in hospitalized children less than 2 years of age, viral load on day 3 of hospitalization was associated with requirement for intensive care and respiratory failure, spotlighting a potential therapeutic window in the hospitalized infant population [25]. In addition to therapies, there is a need for development of cost-effective prophylaxis agents (e.g., novel mAbs and/or antiviral drugs) beyond palivizumab.
